# High-Throughput Resistance Phenotyping of Banana (*Musa* spp.) against *Radopholus similis*

**DOI:** 10.2478/jofnem-2025-0055

**Published:** 2025-12-07

**Authors:** Joseph Kisitu, Kanan K. Saikai, Danny Coyne, Reagan Kanaabi, James Kisaakye, Rony Swennen, Gloria V. Nakato

**Affiliations:** International Institute of Tropical Agriculture (IITA), Kampala, Uganda; International Institute of Tropical Agriculture (IITA), icipe Campus, Nairobi, Kenya; Ceradis B.V., Wageningen, Netherlands; KU Leuven, Department of Biosystems, 3001 Heverlee, Belgium; International Institute of Tropical Agriculture (IITA), c/o Nelson Mandela African Institution of Science and Technology (NM-AIST), Arusha, Tanzania

**Keywords:** banana nematodes, lesion nematode, rapid screening, resistance screening

## Abstract

*Radopholus similis* severely damages banana roots causing significant yield losses. Field screening for resistance is labor intensive and inconsistent due to environmental variation and mixed nematode populations. The screenhouse offers a controlled environment but is limited by the time needed for root development and variation in plant growth. We developed and validated a high-throughput in vitro method for phenotyping banana resistance to *R. similis* using sand-Murashige and Skoog (MS) media. Tissue culture plantlets grown in sterilized sand-MS were inoculated with 50 female *R. similis* after root development and nematodes extracted eight weeks after inoculation to calculate the reproduction factor (RF). Although RF values were higher for in vitro than in the screenhouse, accession responses showed similar trends under both conditions. The in vitro method was rapid, cost-effective with higher throughput, accelerating phenotyping and enabling rapid assessment of banana accessions for breeding programs. Some accessions responded differently to the two methods indicating that additional methods, such as root necrosis scores are important to confirm resistance. This study is the first in vitro-based demonstration of phenotyping for nematode resistance using modified sand-MS media with improved root development and pathogen interactions.

## Introduction

Banana (*Musa* spp.) is an important staple food crop among smallholder farming communities, with a global production estimated at ~196 million metric tons annually (FAOSTAT, 2025), and feeding more than 400 million people. The production of bananas is affected by various constraints, including plant parasitic nematodes (PPN) (Coyne, 2009; Roderick et al., 2012; Viljoen et al., 2017; Sikora et al., 2018). Often, a complex of nematode species, comprising *Radopholus similis*, *Helicotylenchus multicinctus*, *Meloidogyne* spp., and *Pratylenchus* spp., form a community that affects the banana plant (Murongo et al., 2019; Kidane et al., 2020; Nyang'au et al., 2022). This combined community can cause substantial losses (Fogain, 2000; Talwana et al., 2003), amounting to billions of US dollars annually (Elling, 2013). However, *R. similis* is generally considered the most damaging nematode pest of banana (Speijer et al., 2013; Sikora et al., 2018).

Various strategies have been developed to mitigate the issue of nematodes on banana, including cultural, physical, chemical, and biological control; however, the use of plant host resistance offers one of the simplest and most cost-effective mechanisms (Coyne and Kidane, 2018; Sikora et al., 2018; Kisaakye, 2023). This is dependent upon the identification of good, durable resistance, that can be combined with other preferred traits (Backiyarani et al., 2024). Banana breeding pipelines generate hundreds of banana accessions that require phenotyping for nematode resistance. Traditionally, phenotyping has been achieved using whole plants in pots in screenhouses, using either hardened tissue-cultured plants (Speijer and De Waele, 1997) or field-grown suckers (Dochez et al., 2006, 2012), which can limit the size of the trial due to the length of time required and space limitations. The use of high throughput approaches to reduce the time and space required would greatly improve the efficiency of the breeding pipeline (Breeding better bananas, n.d.). Elsen et al. (2002) investigated the use of a gelrite-based Murashige and Skoog (MS) media for in vitro phenotyping of banana resistance against *R. similis*. However, gelrite- or agar-based media do not fully mimic natural conditions, such as aeration and moisture content of the soil. Furthermore, such media do not provide an ideal surface for nematode locomotion and effective root penetration. The current study aimed to evaluate the effectiveness of using tissue-culture (TC) banana plantlets in fine sand and liquid MS under in vitro conditions to phenotype for *R. similis*. The results were compared with the classical approach of screening using pots in the screenhouse.

## Materials and Methods

### TC banana plants

Eighteen banana accessions, currently used as parents in the International Institute of Tropical Agriculture (IITA) banana breeding program, were used (Table 1), including two resistant (Yangambi km5 and SH3142) and four susceptible (Mchare, Valery, Mbwazirume, and Obino l'Ewai) accessions to *R. similis* infection (Pinochet and Rowe, 1979; Dochez et al., 2005, 2009). Plantlets were generated using the shoot-tip culture method (Vuylsteke, 1998) and received at the rooting stage from the IITA TC laboratory, Sendusu, Uganda. Healthy, true-to-type suckers at the bud, peeper, or sword sucker stage were chosen from vigorous, disease-free mother plants. The suckers were washed, outer leaves removed, and corm tissue trimmed to create a 6–8 cm segment that included the shoot apex. The explants were surface sterilized with 15% household bleach containing 1 drop/50 ml Tween 80 for 15 min, followed by 70% ethanol for 5 min, and then 10% bleach with Tween 80 for 25 min. This was followed by three rinses in sterile distilled water (SDW). At the initiation stage, the shoot tip meristem with 3–4 surrounding leaf sheaths was placed upright in propagation medium and incubated in a lighted growth room. To check for bacterial contamination, a small piece of each tissue was placed aseptically onto bacterial growth medium and incubated under light. The plates were observed for 2–3 d to check for fast-growing bacteria and were kept for up to 8 wk to look for slow-growing bacteria. Explants free from contamination were transferred to fresh medium after 2–3 wk. Subsequent transfers were made every 2–3 wk, to remove dead tissue and divide explants to encourage shoot growth. The subculturing into smaller explants was completed every 3–4 wk until shoots developed well-formed pseudostems with at least three leaves. To root the plants, pseudostems were trimmed to 1–2 cm and shoots placed in rooting medium for 3–4 wk to produce 2–5 cm tall plantlets with 3–5 leaves, and well-developed roots suitable for acclimatization. Rooted plantlets were pared under sterile conditions in a laminar flow hood by carefully removing all roots and partially trimming the leaves before use in the in vitro experiments. Additionally, some plantlets were hardened off and these were used in the screenhouse experiments. At 24 wk post initiation, TC-generated plants were ready for the in vitro experiment, while those for the potted experiment were hardened off for 3 mon in the humid chamber and nursery before experimental setup.

**Table 1:** Response of TC banana accessions to *R. similis* infection.

**Accession**	**Genome group**	**Resistance response**
cv. Rose	AA	Unknown
TMB2x 9172	AA	Unknown
SH3142	AA	Resistant
Pahang	AA	Unknown
PITA 7	AAAB	Unknown
PITA 6	AAAB	Unknown
Obino l'Ewai	AAB	Susceptible
Mchare	AA	Susceptible
Mbwazirume	EA-AAA	Susceptible
IITA hybrid 2145/1320	AA	Unknown
716	AA	Unknown
401K-1	AAAA	Unknown
376K-1	AAAA	Unknown
365K-1	AAAA	Unknown
29275S-5	AAAA	Unknown
201087K-4	AA	Unknown
201071K	AA	Unknown
10969S-1	AA	Unknown
Yangambi km5	AAA	Resistant
Valery	AAA	Susceptible

IITA, International Institute of Tropical Agriculture; TC, tissue-culture.

According to the objective and available plants, the number of plants per accession varied by experiment. A significant number of plants were used for experiments on precision and comparison of methods, whereas a smaller number of plants were adopted for feasibility and protocol checks to balance the statistical power.

### *R. similis* culture

The *R. similis population* was originally isolated from the roots of infected banana plants at the IITA station and subsequently maintained as a monoxenic culture on carrot disks (Coyne et al., 2014). The inoculated carrot disks were incubated at 27 ± 0.5°C in the dark to allow for multiplication. Nematodes were subcultured onto fresh carrot disks once every 6–8 wk. When needed, carrot disks were examined under a microscope using ×20 magnification to select disks with live nematodes and then washed off through a sterile 50 mm sieve with SDW into a 5-ml tube, forming a nematode suspension ready for use.

### Plant growth media

**Figure 1:**
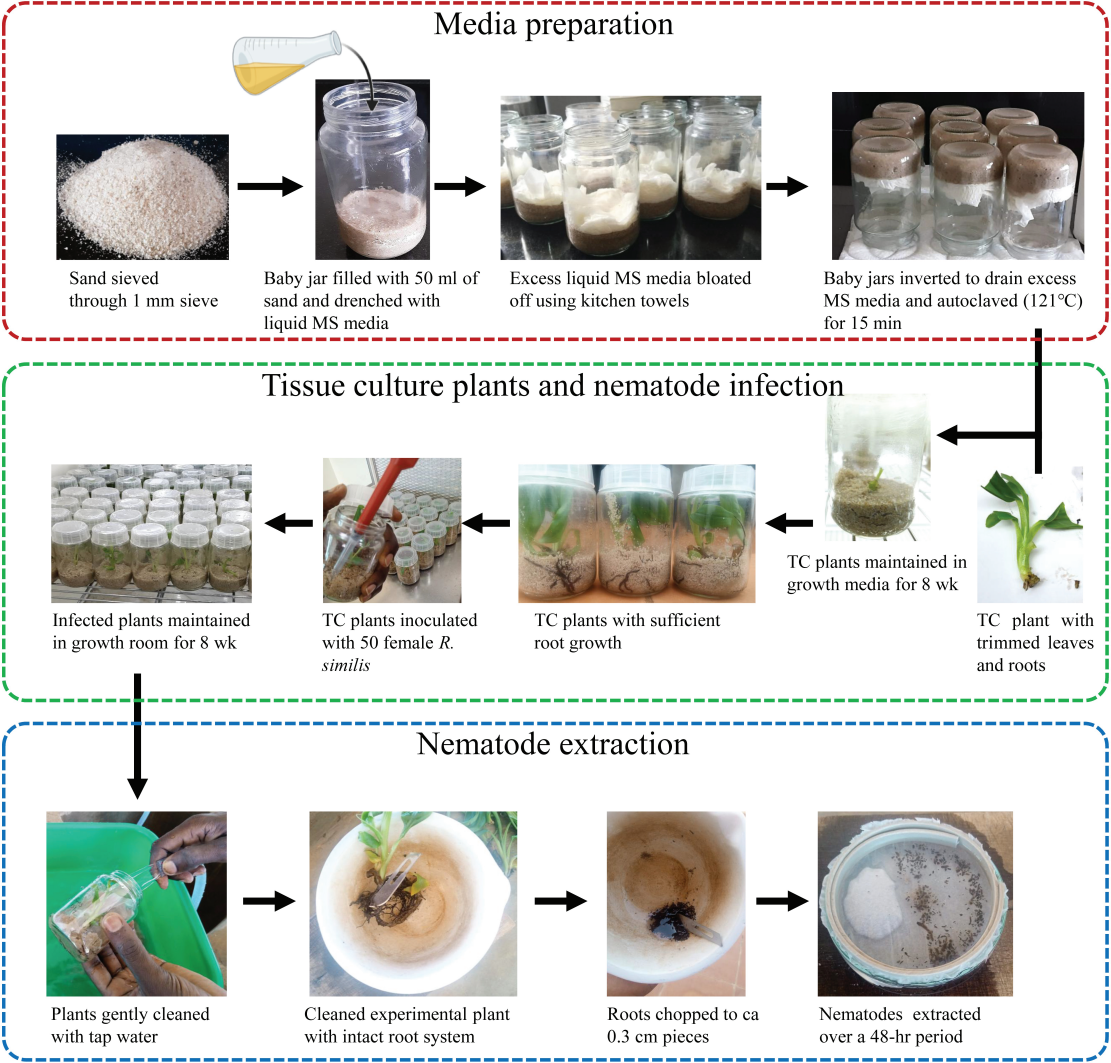
Schematic representation of the methodology used in preparation of modified MS media, nematode inoculation and extraction. MS, Murashige and Skoog, TC, tissue-culture.

The growth media composed of fine river sand with liquid MS media used as a source of nutrients for plant growth. Liquid MS media was prepared according to Murashige and Skoog (1962), with the exclusion of agar and plant growth promoters. The liquid MS media was kept under refrigeration (4 ± 0.5°C) until required. River sand, sourced locally, was sieved through a 1.0 mm aperture sieve, placed in a 10-l bucket, and washed under running tap water until the water flowing from the sand appeared clear. The washed sand was air-dried for more than 48 hr by evenly spreading it on aluminum foil placed on a bench in the laboratory. After drying, 50 ml of sand was placed into 300 ml glass jars, and liquid MS media was slowly and carefully added to each glass jar until all the sand was fully drenched. Each glass jar was gently inverted for 15 min to drain off any excess MS media before placing the lid on. Residual, unabsorbed liquid MS was blotted off using kitchen paper towels, leaving the sand moist but not dripping. The glass jars were then sterilized by autoclaving at 121°C and 15 psi for 15 min. After cooling overnight, a pared TC plantlet was individually and carefully placed in each glass jar using a pair of sterile forceps under a laminar flow hood. The glass jars were individually sealed with lids, wrapped with cling film and labeled, and maintained in the growth room at 25°C for 8 wk following a 16:8-hr dark:light cycle. This period allowed the plants to develop roots in preparation for nematode inoculation (Figure 1).

### Optimization of the in vitro phenotyping protocol for inoculum sterilization

Two susceptible (Valery and Mbwazirume) and two resistant (Yangambi km5 and SH3142) banana accessions were used in the optimization experiments.

The first optimization experiment was conducted to establish the best sterilization method to reduce contamination associated with nematode inoculum in the in vitro studies. This was achieved by comparing streptomycin sulfate solution and SDW as nematode sterilization solutions. The nematode suspension previously prepared was thoroughly homogenized, and a 2-ml nematode suspension was pipetted into each of two 5-ml sterile test tubes. A 6,000 ppm (6 mg/ml) streptomycin sulfate solution was prepared as described by Speijer and De Waele (1997). Nematodes in the 5-ml test tubes were sterilized by adding 1 ml of either streptomycin sulfate solution filtered through a 0.2 mm microfilter using a sterile syringe or SDW. The suspension was left to stand for ~20 min, and the supernatant was carefully pipetted off using a sterile 1 ml pipette, leaving the nematodes at the bottom. This was followed by rinsing three times with SDW, waiting for ~30 min between adding the water and pipetting it off. The nematode density was determined by counting under a compound microscope (×20 magnification) (Leica Microsystems, Wetzlar, Germany), and the final density was adjusted to 250 adult female *R. similis*/ml. The final nematode inoculum was adjusted to pH 5.8, similar to that of the plant growth media.

At 8 wk post establishment of the banana plantlets in the growth media, plantlets with a well-developed root system were selected and used in the bioassay. For each banana accession, eight plantlets were individually inoculated with 200 ml containing 50 *R. similis* females previously sterilized with either streptomycin sulfate solution or SDW under sterile conditions (Elsen et al., 2002). The glass jars with the plantlets were arranged in a completely randomized design (CRD) and maintained in the growth room at 25°C following a 16:8-hr dark:light cycle. The experiment was terminated 8 wk later. The experiment was conducted twice.

**Figure 2:**
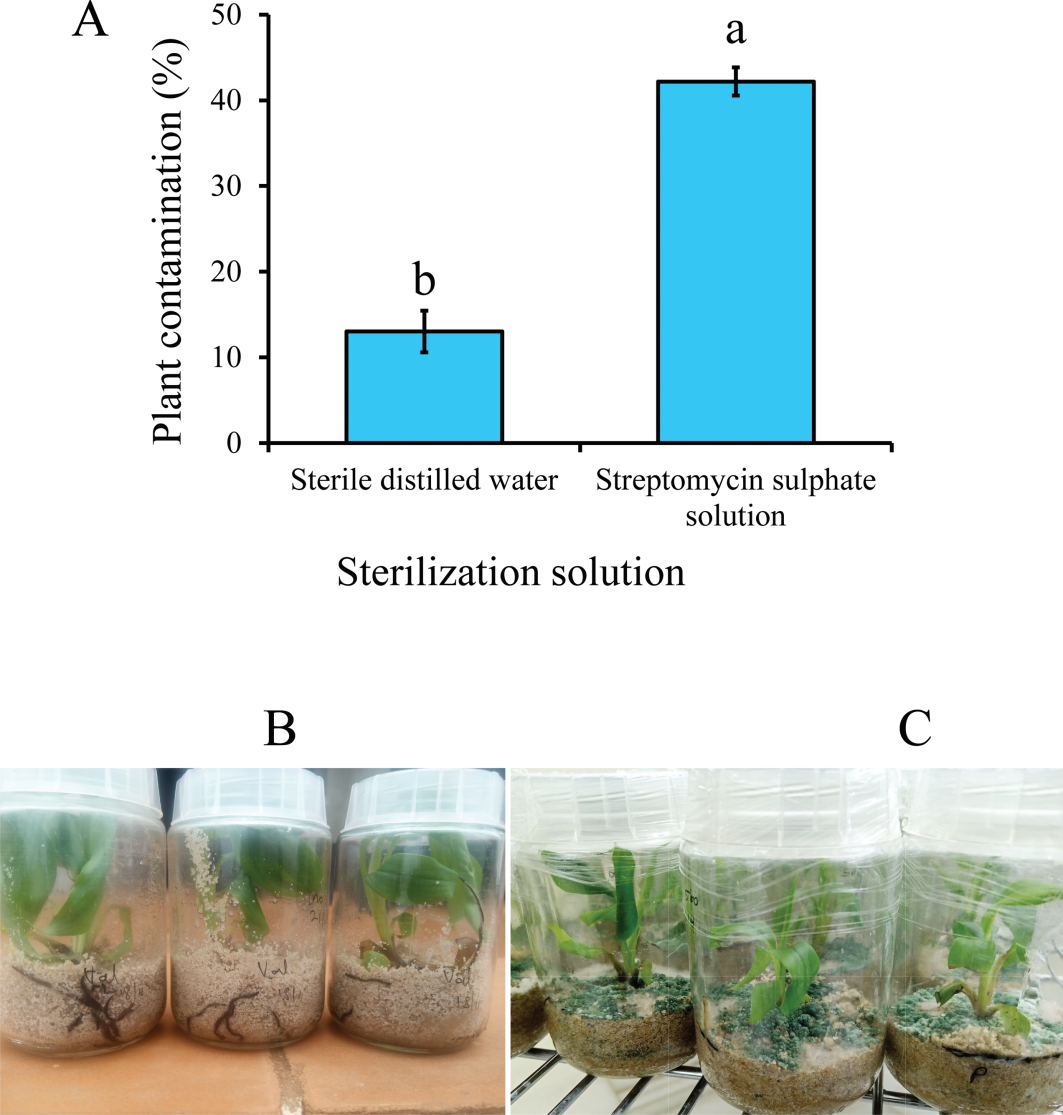
Contamination intensity (A) in banana tissue-cultured plants at 8 wk post-infection with 50 female *R. similis* sterilized with either SDW or streptomycin sulfate solution. Data pooled across two experiments and four banana accessions. A bar graph with different letters indicates a significant difference. Means separated by Tukey's HSD test at *P* < 0.05; Uncontaminated (B) and contaminated (C) plants at 8 wk post infection with *R. similis* sterilized with SDW and streptomycin sulfate solution, respectively. HSD, honestly significant difference; SDW, sterile distilled water.

At experiment termination, the plants were assessed for any signs of contamination. Plant contamination was assessed by observing the growth media for any signs of fungal or bacterial growth (Figure 2C), and the number of contaminated and uncontaminated plants per treatment was recorded. All plants (contaminated or uncontaminated) were carefully removed from the glass jars, and the media was gently washed off using tap water. Roots of individual plants were chopped into ~0.3 cm pieces, and nematodes were extracted from the root material over a 48-hr period using the modified Baermann technique (Coyne et al., 2018; Hallmann and Subbotin, 2018). Nematode suspensions were reduced to 25 ml, and nematode densities quantified from 3 ml × 2 ml aliquots using a compound microscope (×20 magnification). The nematode reproduction factor (RF) was calculated as a ratio of the final (Pf) to the initial (Pi) nematode density of each banana plant.

### Optimization of the in vitro phenotyping protocol for infection time

To establish the optimal time at which to terminate the in vitro phenotyping experiment, 48 plants per accession for two resistant and two susceptible banana accessions were set up as a CRD and maintained in the growth room at 25°C following a 16:8-hr dark:light cycle. Plants were sterilized using SDW, owing to its tested efficacy as a sterilization method from the previous experiment. After inoculation with 50 female *R. similis*, the plants were maintained in the growth room and nematode infection assessed at 2-, 4-, and 8-wk post-inoculation. For each banana accession, eight plants were randomly selected at each time point, and nematodes were extracted and quantified from the whole root system, and RF was determined as previously described. The experiment was conducted twice.

### Validation of the in vitro phenotyping protocol

The validation experiments aimed to confirm consistency in the response of banana accessions to *R. similis* infection under in vitro and screenhouse conditions. This study included all accessions listed in Table 1, with the exclusion of Yangambi km5 and Valery. Mbwazirume and SH3142 were included as susceptible and resistant checks, respectively, owing to their previously established host response to *R. similis* (Talwana et al., 2003; Viaene et al., 2003). In addition, two susceptible landraces were included for additional validation, Mchare and the plantain Obino l'Ewai (Dochez et al., 2009).

A partially replicated design developed using CycDesigN 7.0 software (VSN International Ltd) (Gezan, 2009) with three blocks was adopted for both the in vitro and screenhouse experiments. The experimental arrangement included four plants of each accession per block, with each test accession appearing in two of the three blocks, while the control accessions appeared in all three blocks. Plants in the in vitro experiment were treated as stated in the Section “Optimization of the in vitro phenotyping protocol for infection time”. The in vitro experiment was terminated at 4 wk post-nematode inoculation, the nematodes were extracted, and RF was calculated. The experiment was conducted twice.

For the screenhouse experiment, TC plantlets at the weaning stage were obtained. The roots were gently rinsed free of rooting media using tap water and individually planted in 66 multi-cell plug seedling trays containing a mixture of steam-sterilized topsoil, composted manure, and wood sawdust (3:1:1 v/v). The plantlets were maintained in a humidity chamber (humidity >85%, temperature = 25 ± 2°C) for 4 wk, then transferred to 1-l plastic pots containing the same potting mixture. The plants were maintained in the screenhouse (12:12 h, dark:light) for 8 wk with regular watering. The screenhouse used for maintaining the plants is constructed from a netting material on the side, and the roof is made of a transparent UV-stabilized polythene sheet. Temperature and humidity within the screenhouse were not controlled. During maintenance in the screenhouse, each plant received a biweekly nutrient drench of 150 ml water-soluble NPK (nitrogen, phosphorus, and potassium) (19:19:19) fertilizer (2.5 g/l). Eight weeks after potting, each plant was inoculated with 2 ml of nematode suspension containing 1,000 *R. similis* previously sterilized with SDW. Inoculation with nematodes was performed as described by Coyne and Ross (2014). Nematodes were inoculated in the evening to facilitate their survival. The pots were watered before inoculation and then not watered for 48 hr post-inoculation to prevent nematode loss through flushing, and to allow for nematode penetration. Plants remained in the screenhouse, and the experiment was terminated at 8 wk post-inoculation. Plants were carefully removed from the pot, and the roots of individual plants were gently washed under running tap water. The roots were chopped into ~1 cm pieces and homogenized. A 10 g subsample of the roots was macerated using a laboratory blender (Waring, Torrington, CT) for 15 sec, and nematodes extracted over a 48-hr period using the modified Baermann technique (Coyne et al., 2018; Hallmann and Subbotin, 2018). Nematode RF was determined as previously described.

### Data analysis

All data were analyzed using R (version 4.3.3) (R Core Team) statistical software (R Core Team, 2024). Due to the binary nature of plant contamination data (contaminated versus uncontaminated plants), data were subjected to a generalized linear model (GLM) with binomial distribution and probit link function using the “stats” package to check the main and interaction effects of banana accession and nematode sterilization solution. Model significance was established using analysis of deviance with Wald chi-square test using the “car” package, followed by computation of least-square means, using the “emmeans” package; group means were separated using Tukey's (honestly significant difference [HSD]) multiple comparisons using the “cld” function of the “multcomp” package. Data from the in vitro and pot experiments were analyzed separately by fitting a mixed-effects model using the “lme4” package to evaluate the impacts of banana accession on nematode multiplication, with banana accession specified as a fixed variable, while block, nested within experimental batches, was specified as the random variable. To satisfy the model assumptions, RF data were log_10_(x + 0.1) transformed before analysis of variance (ANOVA). When significant differences were observed between accession means, Bonferroni-adjusted pairwise *t*-tests were employed using the “stats” package, with RF and accession as dependent and independent variables, respectively. Each of the test accessions was individually compared with the resistant (SH3142) and susceptible (Mbwazirume) controls. The RF values were used to generate a forest plot with the standard line indicating the highest RF value at which no significant difference from the resistant check was observed.

## Results

The proportion of contaminated plants was neither influenced by experiment repeat nor banana accession (χ^2^ ≤ 1.02, 1 ≥ *df* ≤ 3, *P* ≥ 0.31). Similarly, there was no significant 2- or 3-way interaction between experiment repeat, banana accession, and nematode sterilization method (χ^2^ ≤ 3.56, 1 ≥ *df* ≤ 3, *P* ≥ 0.25). Consequently, data were pooled across experimental repeats and banana accessions before further analysis. Infecting banana plants with *R. similis* inoculum rinsed with SDW significantly (χ^2^ = 83.64, *df* = 1, *P* < 0.001) reduced the proportion of contaminated plants compared with plants infected with streptomycin-sterilized inoculum (Figure 2).

**Figure 3:**
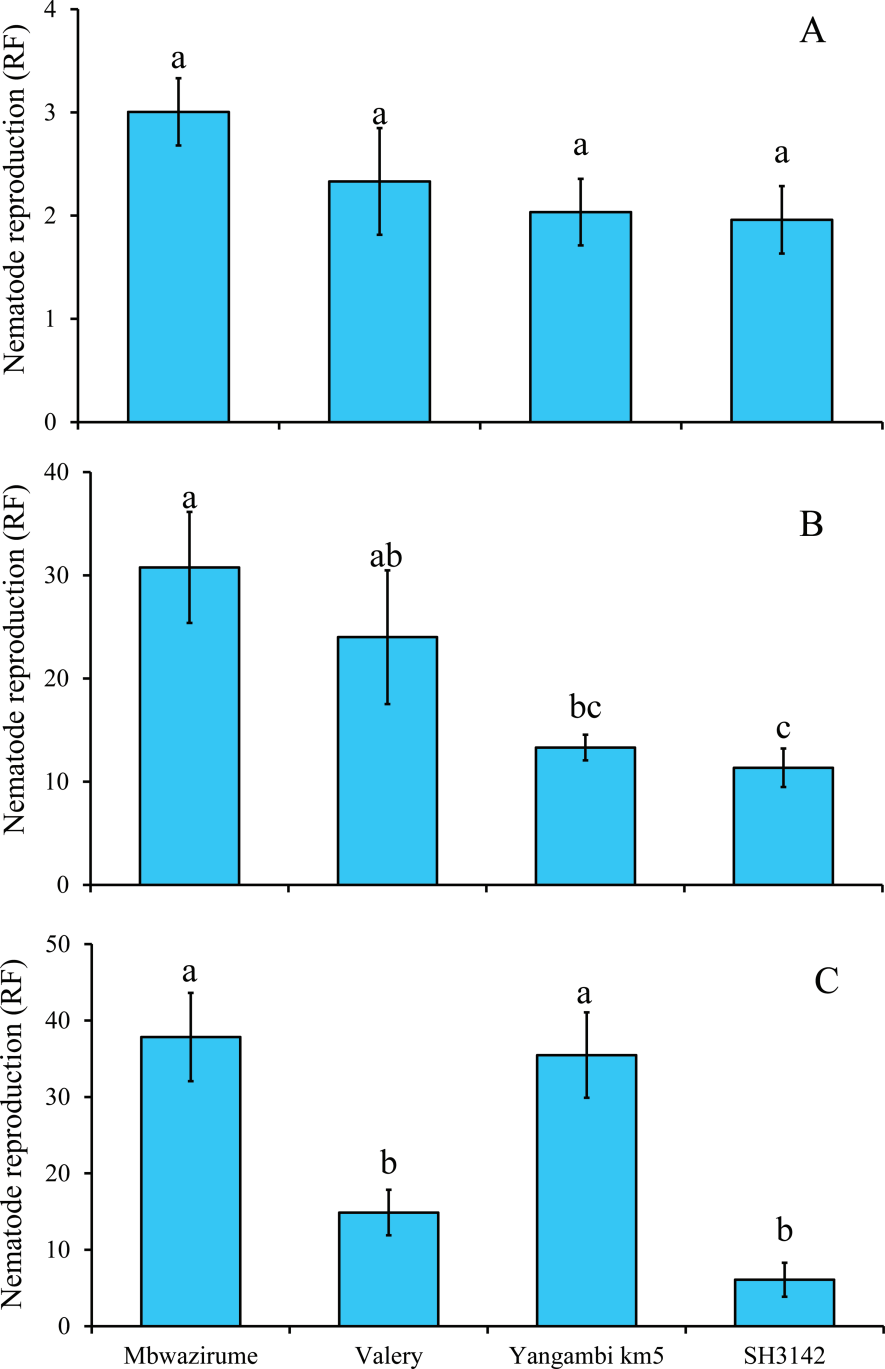
Reproduction of *R. similis* in roots of control banana accessions at 2 wk (A), 4 wk (B), and 8 wk (C) post nematode inoculation under in vitro conditions. At each time point, means followed by different letter(s) indicate a significant difference. Means separated by Tukey's HSD test at *P* < 0.05. HSD, honestly significant difference; RF, reproduction factor.

In the time optimization experiment, RF significantly varied with infection time (χ^2^ = 79.2, *P* < 0.001); overall, the RF (2.3) at 2 wk post-infection of plants with nematodes was lower than the RF (19.9) at 4 wk. However, keeping the plants in the screenhouse longer than 4 wk did not yield any further significant variation in RF values. Similarly, there was a significant (χ^2^ = 38.0, *P* < 0.001) interaction between banana accessions and infection time. Thus, the data were split between time before further analysis. No differences (χ^2^ = 4.6, *P* = 0.21) in RF were observed at 2 wk post-inoculation with nematodes. Differences in infection of banana accessions by *R. similis* became significant at 4- (χ^2^ = 13.2, *P* = 0.004) and 8- (χ^2^ = 37.1, *P* < 0.001) wk post-infection. Consistent results were observed for the susceptible (Mbwazirume) and resistant (SH3142) accessions, respectively (Figure 3).

**Figure 4:**
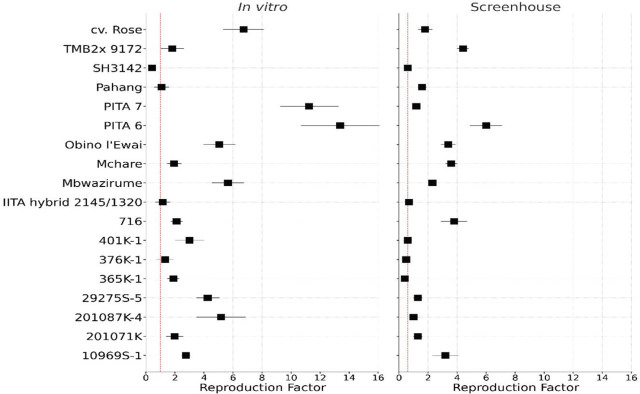
Forest plot comparing nematode reproduction (RF) in banana accessions under in vitro and screenhouse conditions. For each accession, square markers represent the mean RF value, with horizontal standard error bars. The vertical dotted red line indicates the highest RF value at which no significant difference (*P* > 0.05) from the resistant check is observed. RF, reproduction factor.

For the validation experiments, varying levels of nematode reproduction were observed under in vitro and screenhouse conditions. Figure 4 consists of two forest plots comparing the RF of different banana accessions phenotyped under in vitro and screenhouse conditions. The resistant check, SH3142, and susceptible controls, Mbwazirume, Mchare, and Obino l'Ewai, behaved as expected for both the in vitro and screenhouse trials (Figure 4). Lower RF values (RF ≤ 1) indicate resistance to *R. similis*, while higher RF values (RF > 1) are susceptible due to increased nematode reproduction. We observe a similar trend in response to *R. similis* under both screenhouse and in vitro conditions; however, more accessions were observed as resistant in the screenhouse compared with in vitro.

Under both experimental conditions, accessions 376K-1 and IITA hybrid 2145/1320 showed similar levels of resistance as the resistant control, while 716, TMB2x 9172, 10969S-1, 29275S-5, 201087K-4, 201071, cv. Rose, PITA 6, and PITA 7 were susceptible under both experimental conditions. However, accessions 365K-1 and 401K-1 were observed as resistant under screenhouse conditions yet susceptible under in vitro conditions. Conversely, Pahang showed resistance under in vitro conditions, yet was susceptible in the screenhouse (Figure 4). To draw solid conclusions on these three accessions, it is important to refer to root necrosis results from the screenhouse conditions. All accessions phenotyped under in vitro conditions with RF ≤ 1 can be selected for evaluation under screenhouse conditions to confirm their response to *R. similis* infestation.

## Discussion

As shown in Figure 2A, sterilizing nematode inoculum with SDW was effective in reducing contamination of nematode suspensions, thereby providing clean inoculum for in vitro studies. This approach also preserved nematode viability, eliminating the need for antibiotics, reducing competition for nutrients between plant roots and biological contaminants, and ultimately improving plant survival. These findings are consistent with previous studies showing the importance of contamination-free inoculum for reliable host–nematode interaction studies (Speijer and De Waele, 1997; Coyne et al., 2014; Wang et al., 2021). On the contrary, when streptomycin sulfate was applied, there was fungal contamination as shown in Figure 2C, which underscored the limited antimicrobial spectrum and potential to interfere with nematode activity by streptomycin sulfate. Although useful for establishing monoxenic cultures (Coyne et al., 2014), our data showed that streptomycin sulfate is not appropriate for use in sensitive phenotyping experiments.

Accession-specific responses to nematode infestation were more visible at 4- and 8-wk post-inoculation (Figure 3), with 4 wk post-infestation identified as optimal for high-throughput phenotyping. This aligns with nematode biology, where studies indicate that *R. similis* completes its life cycle within 2.5–3 wk at 24–32°C (Marin et al., 1998; Gebremichael, 2015; Chen et al., 2024).

Comparing the response of accessions across the two methods (Figure 4) validated the in vitro assay as a rapid screening tool. Resistant and susceptible controls responded consistently in both methods, although RF values were generally higher under in vitro conditions. This can be attributed to the modified MS medium and fragile root systems of the in vitro plants, which enhanced nematode penetration and reproduction. *R. similis* penetrates its host at the root tip and in root hair zones where the cells are less lignified and more susceptible to invasion (Huang et al., 2019; Chen et al., 2024). The fragile root systems of in vitro plants provided the ideal entry point, thus increasing differences in the accessions. Additionally, the use of sand offered a uniform and aerated growth medium that facilitated nematode invasion of the banana roots. Similar advantages of sand have been reported by Wallace (1966, 1968). Importantly, the use of sand presents a practical solution for resource-constrained research facilities, as it is readily available and easy to work with.

While some accessions exhibited similar responses for both methods, others showed inconsistencies. The relatively higher RF recorded under in vitro as opposed to the screenhouse conditions indicated that nematodes had better access to roots under in vitro conditions due to the confined space and/or uniform pore size of the growth media, while in the screenhouse method, the complex and hardy plant root, and environmental factors such as soil texture and moisture, possibly limited nematode penetration and reproduction. In fact, screenhouse conditions are closer to field conditions, and potentially offer more realistic outcomes for host resistance assessment. Environmental conditions such as root accessibility and nematode survival during the penetration period likely contributed to differences in RF between the in vitro and screenhouse methods.

The in vitro method had a broader range of RF than in the screenhouse, indicating greater sensitivity to subtle differences in resistance. This sensitivity made in vitro a useful method for early-stage, high-throughput screening, where rapid elimination of susceptible accessions is required. However, the screenhouse method provided a more realistic assessment of plant responses under semi-field conditions. In cases where conclusions about resistance remain unclear using either method, we recommend re-evaluating the accession(s) via pot trials and scoring for root necrosis (Speijer and De Waele, 1997), a trait easily scorable in pot trials. Root necrosis data provide additional confirmation of accession responses to nematode infestation (Dochez et al., 2012). It is, however, noteworthy that the use of necrosis as a screening variable is rather subjective compared with nematode reproduction. Additionally, necrosis, particularly arising from a plant's damage-associated immune reactive oxygen species (ROS) burst (Huang et al., 2019), may be highly accession dependent, demeaning comparisons of the rest of the test accessions with included standard controls, unlike nematode reproduction.

For early screening, the in vitro method was efficient, cost-effective, and sensitive for identifying resistance, while the screenhouse method was an essential component of the process. The in vitro method enabled the rapid evaluation of a large number of accessions and an initial bulk screening for selection of potential resistant banana lines, whose resistance can then be confirmed through a pot screening exercise. The in vitro method developed in this study offers clear advantages for rapid, high-throughput phenotyping; the modified growth medium and fragile roots of non-hardened TC-plants may exaggerate nematode penetration and reproduction compared with conditions in the soil. Therefore, although the in vitro method was highly sensitive, it does not capture the complexity of nematode–host interactions under more variable field environments. By contrast, the screenhouse method provided outcomes that closely reflect field reality, such as root hardening, soil structure, and moisture heterogeneity. Nonetheless, the consistency of accession rankings across both methods supports ecological validity. The in vitro method can serve as the first filter, but confirmation in screenhouse remains essential before accessions are advanced in the breeding programs.
